# How the design and complexity of concept maps influence cognitive learning processes

**DOI:** 10.1007/s11423-022-10083-2

**Published:** 2022-01-25

**Authors:** Felix Krieglstein, Sascha Schneider, Maik Beege, Günter Daniel Rey

**Affiliations:** 1 Psychology of Learning with Digital Media, Institute for Media Research, Faculty of Humanities, Chemnitz University of Technology, Chemnitz, Germany; 2 Educational Technology, Institute of Education, Faculty of Arts and Social Sciences, University of Zurich, Zurich, Switzerland; 3 Digital Media in Education, Department of Psychology, University of Education, Freiburg, Germany

**Keywords:** Concept map design, Structuring information, Extraneous cognitive load, Disorientation, Intrinsic cognitive load, Salience

## Abstract

Concept maps are assumed to enhance learning as their inherent structure makes relations between information more salient. Nevertheless, research on how to design concept maps as conducive to learning as possible is still rare. In particular, the salience of spatial arrangement of thematically related concepts within the map as well as the complexity of the map were found to be central design elements that influence learning. This study aimed to examine how the structure (i.e., the salience of the spatial relationship between individual concepts) and the complexity (i.e., number of nodes per sub concept) influence learning. Accordingly, a 2 (low vs. high salience of map structure) × 2 (few vs. many nodes) between-subject design was used (*N* = 122) to examine cognitive processes while learning with a concept map. No significant learning performance differences were found. Concepts maps with a low salience of map structure increased perceptions of disorientation. A serial mediation with learning performances as dependent variable revealed that the salience of the map structure is significantly associated with disorientation and extraneous cognitive load perceptions. By this, current attempts to measure extraneous cognitive load are questioned.

## Introduction

When learning complex information, it is primarily important to be able to transfer the various components into a coherent model. When learners struggle to create such relations mentally, learning may be hindered. In particular, learners with rather low prior knowledge often need further help to internalize knowledge which consists of interconnections. Hereby, different instructional methods offer the possibility to structure and present information in an easy-to-understand way. One way to organize knowledge hierarchically in a rather simple and compact way are concept maps (Cañas et al., 2015; Novak, 1990). In contrast to texts, concept maps represent visualized relationships between thematically-related information units. Therefore, the aim of this study was to gain deeper insights into how the salience of the map structure and the number of nodes per sub-concept affect cognitive learning processes.

### Learning with concept maps

A concept map is defined as “a node-link diagram in which each node represents a concept and each link identifies the relationship between the two concepts it connects” (Schroeder et al., 2018, p. 431) while concepts are illustrated in boxes or oval-shaped forms (Novak & Cañas, 2008). To specify the relationship between two or more concepts, connecting lines are used that can be labeled to further define this connection (Cañas et al., 2015). For instance, the concepts “Facebook” and “Mark Zuckerberg” could be linked with the label “founded by”. The modern idea to structure information in a concept map originates from Novak et al. in the 1970s (Novak & Gowin, 1984). In the literature, similar designations like knowledge maps (O’Donnell et al., 2002), node-link maps (Blankenship & Dansereau, 2000), or mind mapping (Buran & Filyukov, 2015) can be found which deal with the graphical representation of information. Theoretical foundations for the benefit of concept maps can be found in the *assimilation theory of meaningful learning* (Ausubel, 1963). In line with this constructivist approach, meaningful learning only occurs when new ideas and concepts are integrated into already existing knowledge structures (see also Kalyuga, 2009). In line with Mayer (2002), all cognitive processes related to the integration of new information into existing prior knowledge structures can be described as meaningful learning. Learners are therefore considered as active individuals who build up new knowledge on the basis of knowledge already gained (e.g., Bada & Olusegun, 2015). Since their development, concept maps have been examined in numerous learning settings to determine the extent to which they offer an advantage over comparable instructional methods.

In general, the learning-promoting effect of concept maps is meta-analytically supported (Nesbit & Adesope, 2006; Schroeder et al., 2018). In a recent meta-analysis by Schroeder et al. (2018), the learning-beneficial effect could be confirmed with a moderate effect size (*g* +  = 0.58). Hereby, creating concept maps (*g* = 0.72) offered a bigger benefit for learning than studying concept maps (*g* = 0.43). Concept maps can be seen as an effective learning strategy for two main reasons (Schroeder et al., 2018): First, concept mapping promotes meaningful learning. In line with Kalyuga (2009), integrating and organizing new elements into the learner’s knowledge structures can be defined as knowledge elaboration. This process is supported by the inherent structure of concept maps. Therefore, the concept and sub-concept look of concept maps (e.g., Europe—Germany—Federal States—Saxony) illustrates subordinate and superordinate relationships in a more comprehensible way. Compared to texts involving its grammatical structure, concept maps emphasize the macrostructure of the information more clearly (O’Donnell et al., 2002). In this vein, meta-analytical findings from Nesbit and Adesope (2006) revealed that students with low prior knowledge benefitted most from learning with concept maps. Second, the inherent structure of concept maps makes it possible to distribute the cognitive load across the verbal and visual channels of information processing. Thus, a cognitive overload can be avoided (Schroeder et al., 2018; Sweller et al., 2019). Moreover, it is assumed that concept mapping reduces extraneous cognitive processing due to its simpler structure than is the case when studying or writing texts. Concept maps are therefore also beneficial for learners with a low verbal ability (Haugwitz et al., 2010). A recent review by Machado and Carvalho (2020) also indicated that inserting concept maps into university teaching contributes to developing critical thinking skills, promotes meaningful learning, and facilitates student collaboration. In this context, concept maps find wide application in several learning topics. For instance, they are used in chemistry (Talbert et al., 2020), operations management (Essila et al., 2021), and pharmacy courses (Carr-Lopez et al., 2014).

### Cognitive processes while learning with concept maps

While learning several processes take place within the learner. The most important are cognitive processes which determine learning success in a crucial manner. In this vein, the *Cognitive Load Theory* (CLT, Sweller, 2010; Sweller et al., 2019) tries to reconcile human working memory characteristics and the instructional design of multimedia learning environments. Cognitive load can be defined as the cognitive burden which is caused by the learning material in dependence on learners’ prior knowledge (Feldon et al., 2019). Cognitive load subsumes two additive types: intrinsic and extraneous cognitive load (Jiang & Kalyuga, 2020; Sweller et al., 2019). Intrinsic cognitive load (ICL) is determined by task complexity (i.e., the element interactivity) and moderated by learners’ domain-specific prior knowledge (Kalyuga, 2011). The complexity of the learning material is described with the element interactivity on a continuum between low and high. In line with Sweller (2010, p. 124) an element can be defined “as anything that needs to be or has been learned, such as a concept or a procedure”. On the other hand, the prior knowledge may influence the ICL (Chen et al., 2017) as learners with high expertise have already formed schemata, which helps them to solve a problem without a high working memory load. Due to its relevance for learning the ICL can be equated with productive load (Kalyuga & Singh, 2016). In contrast, extraneous cognitive load (ECL) is the burden triggered by information-seeking processes that are caused by a non-optimal design and format of the learning material (Sweller, 2010). Extraneous processing may be also caused when the information is spatially or temporally distributed or not presented in a comprehensible order (van Merrienboer & Ayres, 2005). If working memory resources are already consumed by ECL processes, not enough resources are available to deal with the intrinsic load. The ECL can be changed actively within the design phase of the learning material (Leahy & Sweller, 2016). In line with Kalyuga and Singh (2016) extraneous processing is not relevant for learning and therefore unproductive.

Instructional materials, such as concept maps, also induce a certain amount of cognitive load. Orientated to previous research in the field of educational psychology, it is primarily important to avoid extraneous processing while learning with concept maps to keep free enough working memory capacities for managing the inherent task difficulty (Paas et al., 2003). In line with Tergan (2005), easily comprehensible concept map structures can reduce searching processes, which are detrimental to learning. In this vein, learning with concept maps can suffer from cognitive overload as well as navigational disorientation (Bleakley & Carrigan, 1994). Orienting on Ahuja and Webster (2001), Amadieu et al. (2009) as well as Cress and Knabel (2003), disorientation hinders learning processes in different ways: (1) The learner cannot capture how various concepts within the map are connected; (2) It is more difficult to recognize semantic relationships between the concepts, i.e., which concept is subordinate and which concept is superordinate; (3) The learner is hindered in identifying a path that will function as a guide through the map; and (4) It is sometimes tough to find already read information again. Tergan (2005) assumes that learning scenarios with “ill-structured” content required additional tools to foster learning. One approach is providing a visible hierarchical structure within the concept map (Amadieu et al., 2015). In line with principles of reducing extraneous processing while learning (e.g., *spatial contiguity principle* to prevent learning-hindering split-attention effects; Schroeder & Cenkci, 2018), a visible hierarchical structure within the concept map should lead to better learning performances (DeStefano & LeFevre, 2007; Puntambekar & Goldstein, 2007). Hierarchy results in a high-quality concept map and was thus expected to support learning (Cañas et al., 2015). For this study, an easily identifiable navigation path (adapted from Amadieu et al., 2009) characterizes a salient structure within the map. Therefore, a logical and comprehensible navigation through the learning material is fundamentally conducive to learning, not only in concept maps (Dias & Sousa, 1997).

Another possibility is the implementation of signaling (highlighting relevant information within the learning material; for a meta-analysis see Schneider et al., 2018) which is derived from the CLT. For example, Aguiar and Correia (2016) could show that adding colors into the concept map, in order to group similar information, reduces extraneous cognitive load. Furthermore, Schneider et al. (2021) found empirical evidence for the learning-beneficial effect of implementing organization highlighting principles in concept maps. In this vein, signaling corresponding sub-concepts within the map significantly reduced ECL perceptions. However, the simultaneous usage of these principles (e.g., combining signaling with segmentation) also had negative impacts on learning with concept maps.

To sum up, concept maps were found to have positive effects on learning (Machado & Carvalho, 2020; Nesbit & Adesope, 2006; Novak, 1990; Schroeder et al., 2018). However, empirically documented recommendations on how concept maps should be optimally designed are still rare and require further examinations (e.g., Schroeder et al., 2018).

## The present study

This study investigated how the inherent design and complexity of concept maps can be improved in order to support cognitive processes while learning. Hereby, the effectiveness of concept maps depends to a large extent on the spatial arrangement of the individual sub-concepts, respectively the structure of the entire map. In this vein, Machado and Carvalho (2020) pointed out that students often struggle finding their way through the concept map. Consequently, it may be difficult to integrate the individual sub-concepts and their content into a coherent model.

Also, a salient structured concept map, in which related information is arranged spatially close to each other, should lead to lower ECL and disorientation perceptions. As the saliently structured concept map could promote the learner to find a meaningful reading order (Amadieu & Salmerón, 2014), it is assumed that learners are also more learning-efficient regarding their invested learning time. There might be learner who achieved the same score in a learning test but in a different amount of time.

To summarize, the following hypotheses were formulated:

Learners exposed to a concept map with a high salience of the map structure show …

### H1

better learning performances

### H2

lower ECL perceptions

### H3

lower perceived disorientation

### H4

higher learning efficiency

than learners exposed to a concept map with a low salience of the map structure

In line with findings concerning element interactivity (Kalyuga, 2011), it is assumed that each node of the map can be understood as an element. When thematically and spatially related nodes within the map are assembled to one node, this leads to a lower quantity of elements. When learners are confronted, for example, with three instead of six nodes, they might consider the whole sub-concept as a lower load. The aim is to reduce the element interactivity artificially since the amount of information to be learned remains the same. Just the presentation changes across the factor levels. In a concept map with a higher number of nodes, more separated elements must be connected and learned. Consequently, aggregating thematically related nodes should lead to better learning outcomes. Moreover, when learners are forced to learn with a lower number of nodes, the perceived ICL should decrease because of the reduced element interactivity (Sweller, 2010). In terms of disorientation, a lower number of nodes facilitates learning since additional integrating processes of related nodes are reduced. In a similar way, the artificial reduction lead to higher learning efficiency since less time is required to understand the learning content.

To sum up, the following hypotheses were formulated:

Learners exposed to a concept map with lower number of nodes within sub-concepts show …

### H5

better learning performances

### H6

lower perceived ICL

### H7

lower perceived disorientation

### H8

higher learning efficiency

than learners exposed to a concept map with a higher number of nodes within sub-concepts.

Moreover, a mediation model is proposed under the premise that explicitly the salience of the map structure is associated with the learner's navigational disorientation (DeStefano & LeFevre, 2007; Puntambekar & Goldstein, 2007; Tergan, 2005). Since disorientation is negative for learning it is hypothesized that this perception leads to higher ECL ratings. Following the proposed path, extraneous processing leads to worse learning performance. For this analysis, the retention and comprehension scores were subsumed to the variable learning performance.

### H9

The effect of the salience of the map structure on learning performance is serially mediated by disorientation and extraneous cognitive load.

## Methods

### Design and participants

This experiment is based on a two (salience of the map structure; low vs. high) × two (number of nodes; few vs. many) between-subjects factorial design. An a-priori power analysis (using G*Power; Faul et al., 2007) was conducted with a two-factorial between-subject design with two-factor levels each, a moderate effect size of *f* = .25 (based on meta-analytical findings regarding concept maps and spatial contiguity; Schroeder & Cenkci, 2018; Schroeder et al., 2018), a test power of 1−β = .80 and an error probability of α = .05. This analysis recommended a minimum sample size of *N* = 128. Overall, 130 students from Chemnitz University of Technology, who received either 1-h course credit or the possibility to participate in a voucher lottery, took part in this experiment. Due to technical problems, eight participants had to be excluded. The remaining 122 students (71.3% female; age: *M* = 23.01; *SD* = 3.04) were considered for statistical analyses. Each participant was randomly assigned to one of the four aforementioned treatment groups. Mean prior knowledge was 1.33 (*SD* = 1.19) out of 7 points what can be seen as rather low prior knowledge.

### Instructional material

**Fig. 1:**
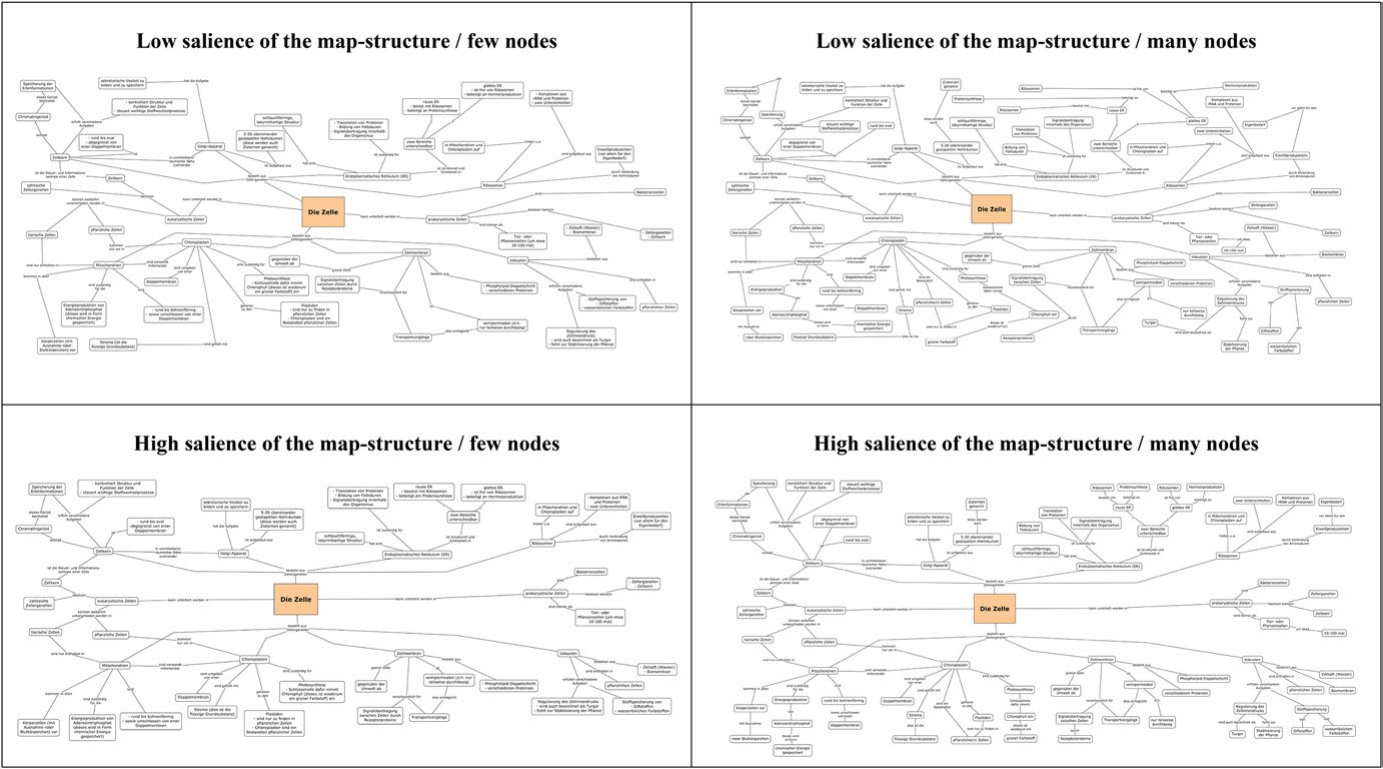
Overview of the four concept maps (conditions) used in the experiment

Two web pages were prepared for the study. The first webpage introduced the participants to the learning content with some general information about the cell and the question of how many cells in the human body exist. By clicking on the forward button, the participants were directed to the second webpage where the concept map was displayed. The concept maps used in this study were developed with the free software tool *CmapTools* (cf. Cañas et al., 2004). Hereby, the maps dealt with biological facts on the cell. More specific, the map presented components of animal and plant cells (eukaryotes) including their formation and functions. Also, prokaryotic cells were briefly stated whereby in particular, the difference between animal and plant cells was emphasized. The concept map was titled “The Cell”. All concept maps comprised of the same amount of information, only the way of presentation was varied across the experimental conditions. To avoid possible *emotional design* effects (Brom et al., 2018), the map was presented on a white background and the font color was black. Just the centrally placed title was displayed in a beige box. Based on the two experimental factors, participants randomly received one map dependent on their condition. An overview of the four concept maps used in the experiment is displayed in Fig. 1.

In terms of the first independent variable, the salience of the structure of the concept map was manipulated. Specifically, a clearly arranged structure should be visible in one condition and an unclear structure in the other condition. The saliently structured concept map leads to easier navigation through the map and to a better understanding of the major and minor components and their semantic relationship. Accordingly, the structure serves as an attention guidance assistant. Lucidly presenting information makes it easier to maintain a meaningful reading order through the concept map. On the other hand, if the map has a less salient structure, it is hardly recognizable how the sub-concepts relate to each other. This is mainly favored by the fact that the individual nodes were distributed as randomly as possible across the map. As a consequence, thematically different nodes are no longer recognizable as such. The learner has to mentally structure the map himself accompanied by many search processes.

**Fig. 2:**
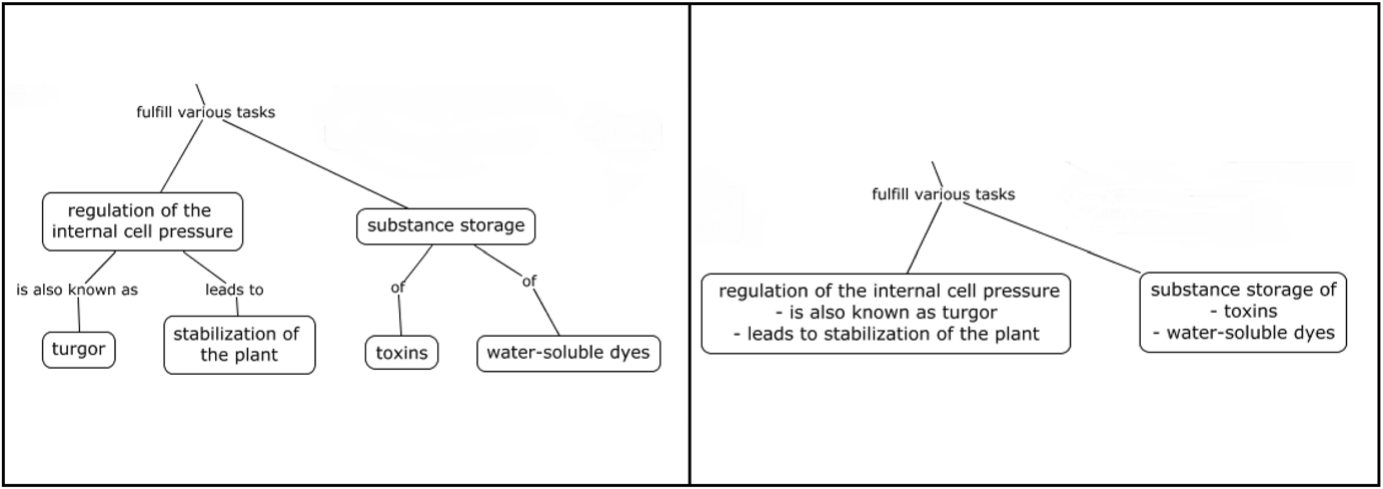
Extract from the concept map (left: many nodes, right: few nodes). *Note*. Instructional material was translated from German to English for this example

Regarding the second independent variable, the two-factor levels differed in the total number of nodes. Concept maps are characterized by the fact that for each node one idea or element is presented. In this study, in the few nodes condition, thematically related nodes (which contains information belonging together) were combined to one node (see Fig. 2) by summarizing corresponding nodes leading to the dissolution of individual nodes.

For instance, the concept “regulation of the internal cell pressure” is connected with the two sub-concepts “turgor” (linked via “is also known as”) and “stabilization of the plant” (link via “leads to”). These two sub-concepts can also be combined in one node due to their thematic proximity. By integrating any nodes that belong together, the total number of nodes could be reduced by 35% (see Table 1).

**Table 1:** Number of nodes for sub-concept of the concept map by condition

Name of sub-concept	Many nodes condition	Few nodes condition
Eukaryotic cells	5	5
Prokaryotic cells	6	4
Nucleus	8	8
Golgi apparatus	4	3
Endoplasmic reticulum	12	6
Ribosomes	6	4
Vacuoles	10	5
Cell membrane	9	6
Chloroplasts	9	5
Mitochondria	8	4
Total number of nodes	77	50

### Measures

For each measure, the reliability indicator Cronbach’s alpha (α) was calculated (Cronbach, 1951; Tavakol & Dennick, 2011) to ensure the internal consistency of the used measurements. In line with Hulin et al. (2001), an alpha-value of 0.6 or more can be considered as satisfactory.

#### Prior knowledge

Learners’ prior knowledge was measured because of its empirically proven influence on cognitive load perceptions and learning performances (Chen et al., 2017). Two different task types were used to capture this concept. First, the open-answer question “What is the difference between eukaryotic and prokaryotic cells?” was given to the participants. A list with correct answers was prepared for evaluation which was conducted by two independent raters. The inter-rater reliability (κ = .92) was almost perfect (McHugh, 2012). The learners could achieve a maximum of three points. For the second task, the participants were asked to assign the following cell organelles to the correct cell type in which they occur: vacuoles, mitochondria, chloroplasts, and Golgi apparatus. Accordingly, four additional points could be reached for this task, whereby a maximum of seven points was awarded in the entire prior knowledge test. Here, no inter-rater reliability was calculated since only one answer per item was correct.

#### Disorientation

For deeper insights on whether the learners were able to navigate through the concept map, modified items of the disorientation scale from Ahuja and Webster (2001) were used (see Table 2). In its original version, this scale is designed to assess the effectiveness of web designs. For this experiment, seven items (α = .93) were adapted for the use of concept maps. Participants had to rate items like “The navigation between the concepts was a problem” on a 7-point scale ranging from (1) “does not apply at all” to (7) “applies completely”.

**Table 2:** Modified Disorientation Scale (Ahuja & Webster, 2001) used in the experiment

Original item in English	Translated item in German	Adapted item in German	Adapted item in English
I felt lost	Ich fühlte mich verloren	Ich fühlte mich beim Lesen der Concept Map verloren	I felt lost while reading the concept map
I felt like I was going around incircles	Ich fühlte mich, als ob ich mich im Kreis drehen würde	Während ich die Concept Map las, fühlte ich mich, als ob ich mich im Kreis drehen würde	As I read the concept map, I felt like I was going around in circles
It was difficult to find a page that I had previously viewed	Es war schwierig, eine Seite zu finden, die ich zuvor angesehen hatte	Es war schwierig, ein Konzept zu finden, welches ich zuvor angesehen hatte	It was difficult to find a concept that I already looked at before
Navigating between pages was a problem	Die Navigation zwischen den Seiten war ein Problem	Die Navigation zwischen den Konzepten war ein Problem	The navigation between the concepts was a problem
I didn't know how to get to my desired location	Ich wusste nicht, wie ich zu meinem gewünschten Ort gelangen konnte	Innerhalb der Concept Map wusste ich nicht, wie ich zu meinem gewünschten Standort gelangen konnte	Within the concept map, I didn't know how to get to my desired location
I felt disoriented	Ich fühlte mich desorientiert	Ich fühlte mich desorientiert beim Lesen der Concept Map	I felt disorientated while reading the concept map
After browsing for a while I had no idea where to go next	Nachdem ich eine Weile geblättert hatte, wusste ich nicht, wohin ich als Nächstes gehen sollte	Nachdem ich die Concept Map eine Weile gelesen hatte, wusste ich nicht, wohin ich als Nächstes gehen sollte	After reading the concept map for a while, I did not know where to go next

These items were answered on a 7-point Likert scale ranging from 1 (“does not apply at all”) to 7 (“applies completely”)

#### Cognitive load

In order to evaluate the impact of the manipulated experimental conditions on learners’ cognitive processes, cognitive load was assessed with a questionnaire from Klepsch et al. (2017). In detail, the German subscales of intrinsic cognitive load (ICL; two items, α = .77, e.g. “For this task, many things needed to be kept in mind simultaneously”) and extraneous cognitive load (ECL; three items, α = .88, e.g., “During this task, it was exhausting to find the important information”) were chosen for this experiment. Each item was rated on a 7-point scale ranging from (1) “not applicable at all” to (7) “fully applicable”.

#### Learning performance

In order to measure the participants’ learning performance, two tests (retention and comprehension) were conducted. For *retention*, which can be defined as remembering (Mayer, 2014), 14 multiple-choice questions were created (α = .74), that questioned knowledge that was explicitly mentioned in the learning material. All questions consisted of four reply options. The number of correct answers differed among all tasks, however, at least one answer was correct. In consequence, the participants got a point if they recognized an item as correct. Besides, one point was awarded when a false item was not selected. For example, the question “What are the functions of the endoplasmic reticulum (ER)?” was given with the answer options (a) “formation of proteins”, (b) “translation of fatty acids”, (c) “signal transmission”, and (d) “storage of genetic information”. Per question, participants could receive a maximum of four points. Overall, 56 points could be maximally achieved by the participants in the retention test.

To measure *comprehension*, four open-format questions were formulated (α = .62). The comprehension tasks served to check to what extent learners understand the learning content and were able to apply the knowledge gained in new situations (i.e., meaningful learning; Mayer, 2002, 2014). For example, two sketchy representations (an animal cell and a bacterial cell) were presented to the participants. Learners had to apply their knowledge of cell structure and components to identify the correct cell type. In another task, learners were asked to explain the possible consequences of a defective cell membrane. To be able to answer this question correctly, participants had to apply their knowledge of the functions of this cell organelle. In sum, 13 points could be reached in the comprehension test.

#### Instructional efficiency

In order to track how efficiently learners used their learning time, efficiency scores were calculated with the following formula (van Gog & Paas, 2008):

$${\text{Efficiency}} = \frac{zP - zT}{{\sqrt 2 }}$$

Learning time (T) and learning performance (P) were z-standardized. After calculation, the efficiency scores ranged from − 2.08 to 1.57 (higher values encode a higher learning efficiency).

### Procedure

Due to the Covid-19 pandemic and related social distancing interventions, the experiment was conducted online via the open-source web conferencing system *BigBlueButton*. Before the experiment, participants got an email with a link to the online room. The instructor informed the participants that they were to learn with a concept map. The participants were also instructed that they would have to answer questions about the learning content after the learning phase. This was to ensure that learners were aware of the goal of the investigation from the beginning. In addition, the participants were asked for informed consent, and instructed to share their screen. This screen sharing was implemented to check whether participants continuously worked with the learning material and the questionnaires and did not check other websites. No personal data was viewed or recorded. Also, participants were instructed to close all tabs except the study website. During the entire experiment, students were able to contact the experimenter either by microphone or chat message in case of problems. The experiment started with the prior knowledge test. After that, students were directed to the website with the learning material. On this website, participants could freely divide their time. Learning time was logged to analyze possible differences. The average duration was 531.34 s (*SD* 339.05 s). After participants’ finished learning, the dependent variables were measured in the following order: (1) disorientation, (2) extraneous und intrinsic cognitive load, and (3) learning tests. In line with ongoing debates that multimedia learning provides rather short-term learning effects in lab experiments (Mayer, 2017), this study tries to examine if the learned information can still be retrieved after an intervention. For this purpose, three filler tasks with rather low cognitive load were implemented. The participants had to name the capitals of different countries, solve geometrical problems and sort confused letters into words. These filler tasks lasted about 5 to 10 min. Afterward, the learning performance was measured. At the end, the participants were asked to provide demographic information such as age, gender, and study subject. Overall, the entire experiment took between 35 and 45 min.

## Results

*IBM SPSS Statistics 27* was used to analyze group differences. For data analyses, multivariate analyses of variance (MANOVAs) and univariate analyses of variance (ANOVAs) were conducted to check for group differences. Follow-up ANOVAs were only calculated if the previously performed MANOVA produced significant effects (Cramer & Bock, 1966). For all variance analyses, the group variables, *salience of the map structure* (low vs. high) and *number of nodes* (few vs. many) were used as independent variables. For the mediation calculation, the SPSS macro *process*, written by Hayes (2013), was used.

Prior knowledge was not included as a covariate since the four groups showed no significant differences (*p* = .17). Besides, there were no significant differences between the four treatment groups in terms of age (*p* = .30) and learning time (*p* = .76). Chi-squared tests revealed no differences with regard to gender (*p* = .95) and subject of study (*p* = .64). Effect sizes for group differences were only reported if they reached statistical significance. Partial eta-squared (η_p_^2^) was used as effect size measure with the conventions .01 for a small, .06 for a moderate, and .14 for a large effect (Cohen, 1988). Correlations between all dependent variables and prior knowledge are displayed in Table 3. In addition, Table 4 shows the mean scores and standard deviations of all dependent variables separated into the four treatment groups.

**Table 3:** Correlations between all dependent variables and prior knowledge

Variables	1	2	3	4	5	6
1. Prior knowledge	–					
2. Disorientation	− .226*	–				
3. Intrinsic cognitive load	− .297**	.545***	–			
4. Extraneous cognitive load	− .219*	.789***	.556***	–		
5. Comprehension	.355***	− .247**	− .179*	− .253**	–	
6. Retention	.192*	− .437***	− .244**	− .381***	.544***	–

*p < .05, **p < .01, ***p < .001

**Table 4:** Mean scores and standard deviations of all dependent variables by experimental group

	Experimental groups							
	Low salience of the map-structure				High salience of the map-structure			
	Few Nodes (*N* = 31)		Many Nodes (*N* = 29)		Few Nodes (*N* = 31)		Many nodes (*N* = 31)	
	*M*	*SD*	*M*	*SD*	*M*	*SD*	*M*	*SD*
Disorientation (1–7)	4.25	1.74	4.38	1.64	3.56	1.58	3.36	1.32
Intrinsic cognitive load (1–7)	5.29	1.25	5.03	1.43	4.94	1.55	4.81	1.17
Extraneous cognitive load (1–7)	4.75	1.76	4.68	1.87	3.99	1.85	4.10	1.62
Prior knowledge (0–7)	1.04	1.13	1.38	1.24	1.22	0.98	1.69	1.35
Retention (0–56)	39.58	6.03	36.66	5.27	37.65	6.24	38.00	6.95
Comprehension (0–13)	5.24	3.16	5.90	2.99	4.40	2.56	5.32	3.38
Learning time	501.51	260.26	496.00	307.93	577.68	339.47	547.87	433.06

The minimum and maximum of each scale are given in parentheses. Learning time is stated in seconds

### Analyses of variance

#### Learning performance

To investigate possible effects of the independent variables on learning performances, a MANOVA was conducted using retention and comprehension as dependent variables. A significant main effect was found for the number of nodes, Wilk’s Λ = .94, *F*(2, 117) = 3.829, *p* = .025, η_p_^2^ = .06. The main effect for salience of the map structure (*p* = .381) and the interaction (*p* = .270) did not reach significance. In terms of retention, a follow-up ANOVA was not able to detect a significant effect for the number of nodes (*p* = .252). For comprehension, the effect for the number of nodes was also not significant (*p* = .155). Consequently, hypotheses 1 and 5 had to be rejected.

#### Cognitive load

For the cognitive load types, a MANOVA was conducted with ICL and ECL as dependent measures. Here, no significant main effect for the salience of the map structure (*p* = .119), the number of nodes (*p* = .627) and for the interaction (*p* = .954) was found. Thus, hypotheses 2 and 6 were also rejected.

#### Disorientation

For perceived disorientation, while learning, an ANOVA was calculated. This analysis found a significant effect of the salience of the map structure; *F*(1, 118) = 8.938, *p* = .003, η_p_^2^ = .07. Accordingly, students in the condition with low salience of the map structure reported their disorientation while learning significantly higher than students in the condition with high salience. This result is in accordance to hypothesis 3. The effect for number of nodes (*p* = .899) as well as the interaction of both factors were not significant (*p* = .560). Accordingly, hypothesis 7 must be rejected.

#### Instructional efficiency

To analyze learning efficiency, an ANOVA was calculated. Hereby, a significant main effect was found for the salience of the map structure; *F*(1, 118) = 4.208, *p* = .042, η_p_^2^ = .03. It indicates that students confronted with a low salience of structure were more efficient in terms of learning time. The main effect for the number of nodes (*p* = .956) and the interaction failed to reach significance (*p* = .100). Based on these results, the hypotheses 4 and 8 had to be rejected.

### Mediation model

More complex models, such as serial mediation, can include more than one mediator (Hayes, 2013). For this study, two mediators (disorientation and ECL) were assumed. A serial mediation was calculated since the two constructs were measured with different questionnaires (Kane & Ashbaugh, 2017). In line with the statistical test assumptions of the mediation (cf. Hayes, 2018), it is assumed that the mediators are in a causal relationship with a temporal precedence.

**Fig. 3:**
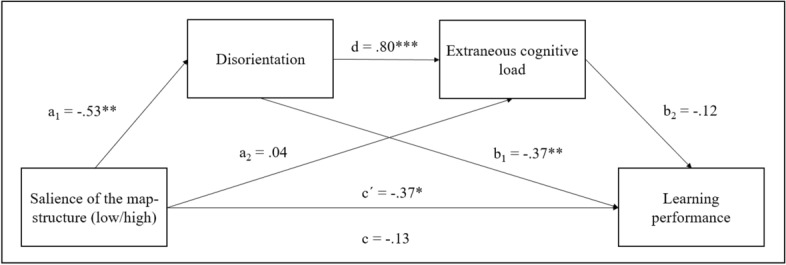
Standardized beta coefficients of the serial mediation analysis paths for the mediating effect of disorientation and extraneous cognitive load on the effect of salience of the map structure and learning performance. *p < .05, **p < .01, ***p < .001

The serial mediation analysis (see Fig. 3) showed that the salience of the map structure had a significant effect on disorientation (a_1_; β =  − 0.53, *SE* = 0.28, *p* = .003). Disorientation, in turn, had a significant effect on ECL (d; β = 0.80, *SE* = 0.06, *p* < .001) and on learning performance (b_1_; β =  − 0.37, *SE* = 0.69, *p* = .007). In addition, the effect of the salience on ECL (a_2_; β = 0.04, *SE* = 0.21, *p* = .722) was not significant.

Moreover, the path from extraneous cognitive load to learning performances was not significant as well (b_2_; β =  − 0.12, *SE* = 0.61, *p* = .371). The total effect of the salience on learning performances did not reach significance (c; β =  − 0.13, *SE* = 1.50, *p* = .489). Interestingly, the direct effect of salience of the map structure on learning performances, controlling for disorientation and extraneous cognitive load, was significant (c’; β =  − 0.37, *SE* = 1.39, *p* = .031), suggesting that the inclusion of the two path variables impacts the effectiveness of the salience of the map structure in terms of learning outcomes. This serial mediation representing a causal chain between salience of the map structure, disorientation, extraneous cognitive load, and learning performances can only be partially confirmed.

## Discussion

### General discussion

The central aim of this study was the experimental verification of two design interventions that play a significant role in the design of concept maps. For this purpose, four different versions of a concept map dealing with a biological topic were given to the participants. These maps differed in terms of the salience of structure and the number of nodes per sub-concept.

Regarding retention and comprehension, the absence of statistically significant effects of the independent variables indicates that it is irrelevant for learning whether the concept map is presented with a low or high salience of the structure or with few or many nodes. From a descriptive point of view, there is hardly any difference between the four groups in terms of the two factors. In terms of the cognitive load facets, the same conclusion can be drawn, since significant effects could not be observed. However, the assumed negative effect of perceived disorientation could be confirmed. When participants were confronted with a difficult-to-encode map (low-salience), they felt more disorientated while learning. Besides the statistical significance of this effect, the explained variance of 7%, which corresponds to a medium effect size, indicates that the map structure affects perceived disorientation notably. Furthermore, navigating between the concepts was complicated by the low salience of the map structure. The number of nodes does not influence perceived disorientation. Concerning the efficiency, learners with the rather unstructured concept map (low salience) were more efficient than learners with a high salience indicating that learners took less learning time to achieve the same performance in the test. Possibly the learning tests were “too easy” so that even short learning times resulted in good performances.

Also, the mediation model showed that a low salience of the map structure significantly affects perceived disorientation. In line with findings from hypertext research (e.g., DeStefano & LeFevre, 2007; Kim & Hirtle, 1995), rather unstructured concept maps caused feelings of disorientation. Problems mainly occur when a high level of disorientation leads to a cognitive overload while learning with concept maps. Following the causal chain, disorientation leads to significantly higher perceptions of the ECL. The high beta coefficient of .80 underlines the strength of this effect. It can be deduced that both disorientation and extraneous processing are a consequence of an inadequate structure within the concept map.

### Implications

After analyzing and interpreting the results of this study, some theoretical and practical implications can be drawn.

#### Implications for practitioners

This study gives some practical insights into designing concept maps in educational settings. Instructional designers should place a primary emphasis on creating concept maps in a way that does not cause feelings of disorientation for the learner. It is particularly important to support learners to find a meaningful reading order through a concept map (Amadieu & Salmerón, 2014). If this prerequisite is met, learners will be able to construct a mental model of both the concept map inherent physical structure as well as the semantic representation (Payne & Reader, 2006). When learning with graphical visualization tools such as concept maps or mind maps, it should be also possible for learners with low prior knowledge to understand how the individual concepts interact.

#### Implications for researchers

On the theoretical side, this study gives a first impulse that disorientation can be regarded as a meaningful supplement of our current understanding of extraneous cognitive load. The current prevailing assumption is that this source of cognitive load is affected by relatively unspecific unfavorable instructional processes and design realizations. Mainly, extraneous load perceptions are recorded in experimental studies using questionnaires. Over time, several validated instruments were developed for measuring the different types of cognitive load along with the ECL (Eysink et al., 2009; Klepsch et al., 2017; Leppink et al., 2013). However, these measurements capture extraneous processing while learning relatively unspecific.

Just the measurement from Eysink et al. (2009) distinguishes the extraneous cognitive load into the dimensions: *navigation*, *design of the learning task,* and *accessibility of information* in order to address the different sources of learning-disrupting factors. Nevertheless, this instrument measures navigation with the question if working within the learning environment was rather easy or difficult. Whether the learner’s navigation impressions while learning are sufficiently captured with an unspecific single item is questionable from a psychometric view. Under the premise that navigation within the material can be seen as a fundamental prerequisite for successful learning, this factor should get more attention in future research. While empirical findings regarding the influence of the structure on concept map effectiveness are still lacking, several studies from the field of hypertext research already examined in which way structure affects learning (e.g., Dee-Lucas & Larkin, 1995; McDonald & Stevenson, 1998). Moreover, disorientation and extraneous cognitive load correlate very highly with each other. The structure in which information is organized crucially affects learning and can be changed by the instructional designer in order to prevent disorientation perceptions while learning. In this vein, the learners perceived navigational disorientation (Amadieu & Salmerón, 2014) could be measured when learning materials display knowledge in a certain spatially and semantic arrangement. For instance, one or more of the following items could be useful, but require factor-analytical examinations (for a beginner’s guide see Yong & Pearce, 2013):

- “It was difficult to get an overview of the structure of the learning material.”
- “The structure within the learning material made it difficult for me to deduce how the individual pieces of information are related”.
- “While learning I had the feeling of getting lost in the learning material.”
- “It was difficult to put the individual pieces of information together to form a big whole.”
- “The structure made it difficult to find important information quickly.”

### Limitations and further directions

From a methodological point of view, it must be noted that the sample is not representative in its composition since participants were mostly female and enrolled in a media-oriented study course. A generalization to other educational settings (e.g., types of schools, school subjects, or students at a different age) is hardly possible and requires replications.

As already mentioned, creating concept maps is associated with a greater learning benefit relative to studying constructed concept maps (Schroeder et al., 2018). In this study, however, participants were asked to learn with prepared concept maps what needs to be considered when interpreting the results of this study. Instructing learners to follow examined design guidelines when creating concept maps on their own can help to increase their knowledge gain.

One of the main goals of this work was to reduce the element interactivity (as one component of the overall ICL) artificially by combining thematically related nodes into one node. This attempt was not successful and is intended to serve as an incentive to become more familiar with possibilities of helping learners to handle a high element interactivity. Besides, the learning material was learner-paced meaning that participants were free in their allocation of learning time. In this vein, a system-paced learning environment could have had a higher impact on the examined effects (Rey et al., 2019).

Besides the learner’s domain-specific prior knowledge, it is also important to consider their knowledge or ability to use the learning medium appropriately. Moreover, one can assume that prior knowledge on the use of concept maps may affect the cognitive load and therefore learning performances. When learners can rely on sufficient knowledge how to use a concept map, it may be easier for them to navigate through the learning material. Consequently, this would also have an influence on the disorientation. Future studies in the field of concept mapping should collect this variable, for example, by means of questionnaires. This would allow learners to indicate their previous experiences (i.e., prior knowledge) with this learning medium.

It must be noted that the concept maps caused high intrinsic loads since a high amount of information was presented. Above all, the condition with many nodes could create a feeling that learners need to internalize as much as possible of the nodes. To be able to accomplish this, sufficient working memory capacities are required. Since this variable was not measured in this study, it cannot be used as an explanatory factor. Further studies should measure the participants’ working memory capacity when learning materials contain memorization tasks (Wilhelm et al., 2013).
